# Analysis of lifestyle and bone mineralization in a population of Spanish young adults

**DOI:** 10.25100/cm.v49i2.2056

**Published:** 2018-09-30

**Authors:** María Correa-Rodríguez, Jacqueline Schmidt-Rio Valle, Ángel Manuel de la Fuente-Vílchez, Blanca Rueda-Medina

**Affiliations:** Departamento de Enfermería. Facultad de Ciencias de la Salud. Universidad de Granada, Granada, España

**Keywords:** Bone density, young adult, exercise, diet, body composition, densidad ósea, adulto joven, ejercicio, dieta, composición corporal

## Abstract

**Objective:**

To analyze the environmental factors (nutritional status, levels of physical activity and nutritional habits) and their possible association with bone mass in a population of young adults.

**Methods:**

The study population consisted of 200 subjects (117 women and 83 men) aged between 18 and 25 years (mean age 20.4 years ±2.2 years). Body composition parameters were measured by an electronic balance (TANITA BC-418MA), nutritional habits were estimated by 72-h dietary recall, level of physical activity was assessed by the International Physical Activity Questionnaire (IPAQ) and bone mass was measured by ultrasonography at the calcaneus.

**Results:**

There were significant differences in bone mass values ​​according to gender (*p*= 0.013). Despite the fact that 70% of the subjects had a body mass index (BMI) within the normal range, 20% had overweight or obesity. 49% of the individuals had a moderate level of physical activity, although women had lower levels of physical activity than men (17.9% vs 8.4%). Most diets among young adults were hyperprotic, hyperlipidic and low-carbohydrates, with low-calcium and fiber intakes and high-phosphorus. Analysis of bone mass according to BMI showed higher values ​​as the weight category rises, with significant differences in women. Higher values ​​of bone mass were shown as the level of physical activity was higher, showing significant differences in males.

**Conclusion:**

Our results show that BMI and level of physical activity are significantly associated with bone health in a population of young adults, suggesting the relevance of promoting healthy lifestyles as a strategy for the early prevention of osteoporosis.

## Resumen

**Objetivo:**

Analizar los factores ambientales (estado nutricional, nivel de actividad física y hábitos nutricionales) y su posible relación con los niveles de masa ósea en una población de adultos jóvenes.

**Métodos:**

La población de estudio estuvo constituida por 200 sujetos (117 mujeres y 83 hombres) de edades comprendidas entre 18 y 25 años (edad media 20.4 años ± 2.2 años). La composición corporal se evaluó con balanza electrónica (TANITA BC-418MA), la valoración de los hábitos nutricionales mediante Recordatorio de 72 h, el nivel de actividad física a través del Cuestionario Internacional de Actividad Física (IPAQ) y la masa ósea mediante ultrasonografía en el calcáneo.

**Resultados:**

Existen diferencias estadísticamente significativas en los valores de masa ósea en función del sexo (*p*= 0.013). A pesar de que el 70% de los sujetos presentaron un índice de masa corporal (IMC) dentro de los límites de la normalidad, un 20% padece sobrepeso y obesidad. El 49% de los individuos tenian un nivel moderado de actividad física, aunque las mujeres presentaron niveles más bajos de actividad física que lo varones (17.9% vs 8.4%). Las dietas mayoritarias entre los adultos jóvenes son hiperprotéicas, hiperlipídicas y bajas en hidratos de carbono, observándose una ingesta baja de calcio y fibra y alta en fósforo. El análisis de la masa ósea en función de IMC mostró valores mayores conforme asciende la categoría ponderal, existiendo diferencias significativas en mujeres. Igualmente, se muestran valores más altos de masa ósea conforme mayor es el nivel de actividad física, evidenciando diferencias significativas en los varones.

**Conclusión:**

Nuestros resultados muestran que el índice de masa corporal y el nivel de actividad física se asocian significativamente con la salud ósea en una población de adultos jóvenes, evidenciando la importancia del fomento de estilos de vida saludables como estrategia para la prevención temprana de la osteoporosis.

## Introduction

Osteoporosis, considered as a global health problem, is defined as a systemic skeletal disorder characterized by low bone mass and deterioration of the microarchitecture of bone tissue, with the consequent increase in bone fragility and greater susceptibility to fractures ^1^. Bone mass is the result of the maximum amount of bone reached during growth, known as peak bone mass (PBM), which is subtracted from the amount of bone that has been lost ^2^. The acquisition of PBM depends on environmental and genetic factors ^3^ ^,^ ^4^. Among the modifiable factors, body weight, physical activity and nutrient intake have been shown to influence bone mass throughout life ^5^.

Previous studies have shown significant associations between high levels of physical activity and higher values ​​of bone mass at early ages ^6^ ^-^ ^9^. It has also been reported that physical activity improves bone mineralization due to the increase in size both in cortical and trabecular portions of bone in those subjects who increase physical activity ^10^. In contrast, in sedentary individuals the inverse effect has been observed ^11^. The increase in physical activity provides greater bone strength, as well as a protective effect against fractures by improving and preserving the elastic properties of bone ^12^.

Regarding nutritional habits, although previous works have shown the relevance of an adequate calcium intake during childhood and adolescence for the prevention of osteoporosis ^13^ ^,^ ^14^, some studies indicate that this benefit in bone mineralization it is not fully conclusive ^6^ ^,^ ^15^ ^,^ ^16^. On the other hand, the excessive intake of phosphorus may cause an imbalance in the calcium-phosphorus index that leads to an increase in bone resorption and fragility, as well as a decrease in the achievement of PBM ^17^.

Nowadays, the increase in overweight and obesity rates during childhood and adolescence is one of the most prevalent health problems ^18^ ^,^ ^19^. Studies that focus on the role of overweight and obesity in bone mass at early ages have evidenced contradictory results, reporting both positive and negative effects ^20^ ^-^ ^24^.

The non-acquisition of optimal PBM during growth will increase the risk of osteoporotic fractures later in life^25^. Taking into account that the acquisition of bone mass depends on environmental and genetic factors, it is of especial interest to investigate the profile of young adults in terms of levels of physical activity, nutritional status and nutritional habits. In this context, the aim of this study was to evaluate the environmental factors (nutritional status, levels of physical activity and nutritional habits) and their possible association with bone mass in a population of young adults.

## Materials y Methods

### Design and study population Subjects

This was a descriptive cross-sectional study. The study population consisted of 200 young adults (117 women and 83 men) aged 18 to 25 from some educational centers of the province of Granada (Spain). The inclusion criteria for participation in the study included the signed informed consent and an age between 18 and 25 years. Regarding the exclusion criteria, subjects with medical illnesses and/or disorders that could affect bone mineralization (traumatic, metabolic or systemic disorders affecting central nervous system, autonomic, endocrine or severe psychopathological disorders) and those who were in treatment with drugs that influence bone mineralization were excluded. The study was approved by the Human Research Ethics Committee of the University of Granada (nº 78/CEIH/2015) (n= 384) and all volunteers were recruited after signing the informed consent document. The study protocol followed the ethical guidelines and principles for medical research in humans in accordance with the Declaration of Helsinki.

### Anthropometric assessment

Body height was measured with a stadiometer to the nearest 0.1cm (Holtain 602VR^®^) and recorded in centimetres. Body composition parameters were assessed using an electronic balance (TANITA BC-418MA^®^), to the nearest 100 g. BMI (Body Mass Index) was calculated as weight over height squared (kg/m^2^). BMI-value was classified according to the recommendations of the World Health Organization (WHO) as underweight (BMI <18.5), normal-weight (BMI= 18.5-24.9), overweight (BMI= 25.0-29.9) and obesity (BMI ≥30).

### Bone mass assessment

Bone mass status was assessed by ultrasonography at the calcaneus using the CUBA PLUS v4.1.0 bone densitometer (McCue Ultrasonics Limited, Compton, Winchester, UK). The parameter of bone mass provides by this device is the Broadband Ultrasond Attenuation (BUA) measured in dB/MHz. Bone assessment by ultrasonography is a technique used in pediatrics for its reproducibility (99%), accuracy (1-2% error), short time (5-10 min) and radiation-free ^26^.

### Nutrient intake

Dietary intake was assessed using a 72-h dietary recall. Completed food records were analyzed using a computerized nutrient analysis program (Nutriber 1.1.5). To assess micronutrients intakes, normal intakes were considered as calcium intake between 900 and 1,200 mg/day ^27^, fiber intake between 20 and 30 mg/day and phosphorus intake between 600 and 800 mg/day ^28^.

### Physical activity

Physical activity was assessed using the International Physical Activity Questionnaire (IPAQ) ^29^. It is a validated tool for measuring physical activity in adult population. The unit of measurement is MET (Metabolic Equivalent= 3.5 mL of O_2_/kg of weight/min) and it is estimated considering intensity of physical activity x minutes x days per week. Based on MET calculation, levels of physical activity were classified as low, moderate and high.

### Statistical analysis

Numerical variables were expressed as mean ± standard deviation (SD) and nominal variables as percentages and frequencies. Differences in dietary intake according to gender were assessed through independent t-test where normally distributed. The chi-squared test was used to determine differences between categorical variables. Differences of BUA values ​​according to BMI categories and level of physical activity were determined using analysis of variance (ANOVA). The cut-off value for significance was set as *p* <0.05. All analyses were performed using SPSS version 20.0 (SPSS, Chicago, IL, USA).

## Results

### Anthropometric characteristics, physical activity and bone mass

The mean age of the study population was 20.4 ±2.2 years. The anthropometric characteristics, levels of physical activity and bone mass of the study population stratified by gender are shown in Table 1. Based on the BMI classification, 70% of the individuals (70.1% women and 69.9% men) were of normal weight. The prevalence of obesity and overweight in the total population was 20% and 10% of the young adults had underweight. 

**Table 1:** Anthropometric characteristics, physical activity and bone mass of the study population.

	Women (n= 117)	Men (n= 83)
	Mean ±SD	Mean ±SD
Age (years)	20.10±2.06	20.76±2.40
Height (m)	1.64±0.06	1.76±0.07
Weight (kg)	58.75±10.22	73.69±12.27
Fat mass (kg)	14.43±7.09	11.53±6.26
Lean mass (kg)	44.32±3.73	62.12±7.73
**BMI (%)**		
Underweight	17.0(14.5)	3.0 (3.6)
Normal-weight	82.0 (70.1)	58.0 (69.9)
Overweight/Obesity	18.0 (15.4)	22.0 (26.5)
**Level of physical activity** (%)		
Low	21.0 (17.9)	7.0 (8.4)
Moderate	64.0 (54.7)	34.0 (41.0)
High	32.0 (27.4)	42.0 (50.6)
**Ultrasound measurements in the calcaneus**		
BUA (dB/MHz)	82.8±16.3	97.9±16.5

SD: standard deviations; BUA: Broadband ultrasound attenuation.

14% of the subjects had a low level of physical activity, 49.0% moderate and 37.0% high. The prevalence of high physical activity was higher in men (50.6%) compared to women (24.7%). Similarly, note that only 8.4% of men had low levels of physical activity whereas the prevalence in women was 17.9%.

The mean calcaneus BUA for the population was 89.07 ±17.93 dB / MHz. Women had significantly lower BUA values than males (82.83 ±16.29 vs 97.87 ±16.45; *p*= 0.013) (data not shown).

### Nutritional habits

An analysis of nutritional habits was conducted according to gender including macronutrients (carbohydrates, proteins and lipids) and micronutrients (calcium, phosphorus and fiber) intakes (Table 2). No statistically significant differences were found in dietary patterns between men and women (data not shown). Note that imbalances in energy intake, macro and micronutrient intakes were observed. Most prevalent diet was hyperphotheic and hyperlipidemic and calcium and fiber intakes were below the recommendations in both gender. In contrast, a high intake of phosphorus was identified.

**Table 2:** Nutritional habits according to gender.

Intakes	Women		Men	
	n	%	n	%
Energy				
Hypocaloric Diet	63	55.3	57	68.7
Normocaloric Diet	20	17.5	9	10.8
Hypercaloric Diet	31	27.2	17	20.5
**Carbohydrates**				
Low carbohydrate Diet	61	52.6	39	47.0
Normal-carbohydrate Diet	17	14.7	19	22.9
High carbohydrate Diet	38	32.8	25	30.1
**Proteins**				
Hypoproteic Diet	2	1.7	1	1.2
Normal-hypoproteic Diet	40	34.5	28	33.7
Hiperproteic Diet	74	63.8	54	65.1
**Fats**				
Hypolipid Diet	44	37.9	27	32.5
Normal-lipid Diet	20	17.2	26	31.3
Hiperlipid Diet	52	44.8	30	36.1
**Calcium**				
Low-calcium Diet	74	63.8	47	56.6
Normal-calcium Diet	24	20.7	18	21.7
High-calcium Diet	18	15.5	18	21.7
**Phosphorus**				
Low-phosphorus Diet	3	2.6	1	1.2
Normal- phosphorus Diet	13	11.2	3	3.6
Normal- phosphorus Diet	100	86.2	79	95.2
**Fiber**				
Low-fiber Diet	77	66.4	47	56.6
Normal-fiber Diet	18	15.5	23	27.7
High-fiber Diet	21	18.1	13	15.7

No statistically significant differences were observed according to gender.

### Body mass index, level of physical activity and bone mass

Bone mass according to BMI categories (underweight, normal-weight and overweight / obesity) and levels of physical activity (low, moderate and high) are shown in Table 3. Statistically significant differences were observed in BUA values between underweight and normal-weight subjects (*p*= 0.001), normal-weight and overweight / obesity (*p*= 0.001) and underweight and overweight / obesity (*p* <0.001) in women. In men, no significant differences were found. Regarding the level of physical activity, statistically significant differences were found between subjects with low and moderate levels of physical activity (*p*= 0.021), and with low and high levels of physical activity (*p*= 0.015) in men.

**Table 3:** Bone mass according to BMI and level of physical activity.

	Women (n= 117)			Mean (n= 83)		
	BMI					
	Underweight (n= 17)	Normal-weight (n= 82)	Overweight/Obesity (n= 18)	Underweight (n= 3)	Normal-weight (n= 58)	Overweight/Obesity (n= 22)
	Mean ±SD	Mean ±SD	Mean ±SD	Mean±SD	Mean ±SD	Mean ±SD
BUA (dB/Mhz)	^a^69.47±13.71	^b^82.65±13.73	^c^101.38±18.57	87.33±16.80	96.10±14.95	102.82±19.85
	**Level of physical activity**					
	Low (n= 21)	Moderate (n= 64)	High (n= 32)	Low (n=7 )	Moderate (n= 34)	High (n=42)
BUA (dB/Mhz)	79.33±15.94	84.48±17.30	81.81±14.36	^d^87.00±9.16	98.79±17.97	^e^98.93±15.73

SD: Standard deviations; BUA: Broadband ultrasound attenuation.

^a^Underweight vs Normal-weight *p* <0.001.

^b^Normal-weight vs Overweight/obesity *p*<0.001

^c^Overweight/obesity vs Underweight *p* <0.001.

^d^Low vs moderate *p* <0.05.

^e^Low vs high *p* <0.05.

## Discussion

The profile of young adults regarding environmental factors that determine bone mass acquisition is limited. In the present study, the nutritional status, nutritional habits, level of physical activity and bone status were investigated in 200 young adults. Our results showed that BMI and level of physical activity are significantly associated with bone health in a population of young adults, suggesting that both are predictor factors of bone status at early ages.

Nutritional status data showed a higher tendency to overweight and obesity in males (26.5%) compared to women (15.4%). In contrast, the prevalence of underweight was higher in women compared to men (14.5% versus 3.6%). Similar trends were observed in the National Health Survey published in Spain ^30^, supporting that the prevalence of obesity and overweight is higher among men. Similarly, there is also a higher tendency to underweight in women compared to men.

With regards to physical activity, men had higher levels of high physical activity than women (50.6% versus 27.4%.) In relation to moderate physical activity, the prevalence was similar in both gender (41.0% in males versus 54.7% in females). However, the prevalence of low physical activity was notably higher in women compared to men (women 17.9% versus men 8.4%). It should be noted that in our study population the prevalence of high level of physical activity (37.6%) was higher compared to National Health Survey data, which reported that four out of ten subjects are sedentary ^30^.

Nutritional patterns observed in this study population reveal that intake of lipids and proteins were higher compared to recommendations whereas carbohydrates intake was lower. Similarly, we found that the majority of young adults had a low energy intake. Our results are consistent with those obtained in previous studies conducted in Spanish populations ^31^ ^-^ ^34^.

The mean bone mass value for the total population was 89.07 ±17.93, similar to that observed for young adults in previous studies ^35^ ^-^ ^37^. A significant difference in bone mass was found according to gender, reporting that women has significantly lower bone mass values than men. These differences have been also previously reported ^37^ ^,^ ^38^, coinciding with the high prevalence of osteoporosis in women during adulthood ^39^. These findings might support that the influence of hormonal and genetic factors in osteoporosis might play main roles at early ages.

In agreement with previous studies ^23^ ^,^ ^24^ ^,^ ^31^, we reported that bone mass is linked to BMI since higher BUA values were observed as the BMI category increases (underweight, normal-weight and overweight/obesity) in both gender. The lack of significant differences in males might be due to the limited sample size or the observed differences regarding nutritional status between both gender. 

Additionally, in line with previous studies ^6^ ^,^ ^7^, our findings showed significant differences in bone mass values according to the level of physical activity (low, moderate and high) in males, reporting higher BUA values in the calcaneus as greater is the level of physical activity. The fact that we only identified significant results in men might be due to the remarkable differences in the level of physical activity between both groups.

Regarding limitations of the study, it should be highlight its cross-sectional design that limits the ability to determine causal relationships. In addition, other potential limitation would be inherent to the assessment of physical activity using a self-administered questionnaire. However, it is important to note that IPAQ has been widely established as a validated tool for estimating physical activity ^29^. Finally, the fact that only young adults were included in the study population could limit the generalizability of the results to other populations.

## Conclusion

Our results show that BMI and level of physical activity are significantly associated with bone health in a population of young adults, suggesting the relevance of promoting healthy lifestyles as a strategy for the early prevention of osteoporosis. It would be of special interest to carry out future studies investigating the role of genetic factors on bone mass during early adulthood, since it has been established that they can influence up to 80% in the acquisition of the PBM. 
