# Supplementary material for: Analysis of lifestyle and bone mineralization in a population of Spanish young adults
Source: Colomb Med (Cali). 2018 Sep 30;49(3):213–8. doi: 10.25100/cm.v49i2.2056 (PMC6220484; doi:10.25100/cm.v49i2.2056)
Supplement: Related texts

# Research article: Análisis de los estilos de vida y la mineralización ósea en una población de adultos jóvenes españoles

## Introducción

La osteoporosis, considerada como un problema sanitario a nivel global, se define como un trastorno esquelético sistémico caracterizado por baja masa ósea y deterioro de la microarquitectura del tejido óseo, con el consecuente incremento de la fragilidad ósea y una mayor susceptibilidad a las fracturas ^1^. La masa ósea del individuo es el resultado de la cantidad máxima de hueso que ha alcanzado durante su crecimiento, conocido como pico de masa ósea (PMO), a la que se le resta la cantidad de hueso que se ha perdido desde ese momento ^2^. La adquisición del PMO depende de factores ambientales y genéticos ^3^^,^^4^. Entre los factores modificables que influyen en el nivel de mineralización ósea del individuo a lo largo de su vida se encuentran la masa corporal, la actividad física y la ingesta de nutrientes ^5^.

Estudios previos han mostrado una asociación significativa entre niveles altos de actividad física con valores mayores de masa ósea en edades tempranas ^6^^-^^9^. Además se ha evidenciado que la actividad física provoca un incremento en el nivel de mineralización ósea debido al aumento del tamaño tanto en la porción cortical como en la trabecular del hueso en aquellos sujetos que aumentan la actividad física ^10^. Por el contrario, en los individuos que disminuyen o no realizan actividad física, se observa el efecto inverso ^11^. El incremento de la actividad física proporciona mayor fortaleza ósea, así como un efecto protector frente a fracturas al mejorar y conservar las propiedades elásticas del hueso ^12^.

Respecto a los hábitos nutricionales, a pesar de que estudios previos han mostrado la relevancia de una adecuada ingesta de calcio durante la niñez y la adolescencia para la prevención de la osteoporosis ^13^^,^^14^, diversos estudios señalan que dicho beneficio en la mineralización no queda evidenciado en su totalidad ^6^^,^^15^^,^^16^. Por otro lado, la excesiva ingesta de fósforo produce un desequilibrio en el índice calcio-fósforo que conlleva el aumento de la resorción y fragilidad ósea, así como una disminución en la consecución del PMO ^17^.

En la actualidad, el aumento en las tasas de sobrepeso y obesidad durante la infancia y la adolescencia es uno de los problemas de salud más frecuentes^18^^,^^19^. Los estudios que se centran en el papel del sobrepeso y la obesidad en la masa ósea a edades tempranas han mostrado resultados contradictorios, evidenciando tanto efectos positivos como negativos ^20^^-^^24^.

La no adquisición del PMO óptimo durante el periodo de crecimiento incrementará el riesgo de sufrir fracturas osteoporóticas en la senectud ^25^.Teniendo en cuenta que la adquisición de la masa ósea depende de factores ambientales y genéticos resulta trascendental conocer el perfil de los adultos jóvenes en cuanto a su nivel de actividad física, estado nutricional y hábitos nutricionales. En este contexto el objetivo del presente trabajo fue evaluar los factores ambientales (estado nutricional, nivel de actividad física y hábitos nutricionales) y su posible asociación con el nivel de masa ósea en una población de adultos jóvenes. 

## Materiales y Métodos

### Diseño y población de estudio Sujetos

El diseño del estudio fue transversal y descriptivo. La población de estudio estuvo constituida por 200 adultos jóvenes (117 mujeres y 83 hombres) de edades comprendidas entre 18 y 25 años pertenecientes a varios centros educativos de la provincia de Granada (España). Los criterios de inclusión para la participación en el estudio incluyeron la firma previa del consentimiento informado y una edad comprendida entre los 18 y 25 años. Respecto a los criterios de exclusión, se excluyeron del estudio aquellos sujetos que hubiesen presentado o presentaban enfermedades médicas y/o trastornos que pudiesen afectar al nivel de mineralización ósea (trastornos traumáticos, metabólicos o sistémicos que afecten al sistema nervioso central, autónomo, endocrino o trastornos psicopatológicos severos) y aquellos que se encontraban en tratamiento con fármacos que interfieran en la mineralización ósea. El estudio fue aprobado por el Comité de Ética de Investigación Humana de la Universidad de Granada (n= 384) y todos los voluntarios se reclutaron tras mostrar su consentimiento por escrito. El protocolo del estudio siguió las directrices y principios éticos para las investigaciones médicas en seres humanos según se recoge en la Declaración de Helsinki. 

### Valoración antropométrica

La altura corporal se midió con un estadiómetro a 0.1cm (Holtain 602VR^®^) y se registró en centímetros. La composición corporal se midió con una balanza electrónica (TANITA BC-418MA) con una precisión de ±100 g. El IMC (Índice de masa corporal) fue calculado como el peso corporal dividido por la altura al cuadrado (kg/m^2^). Siguiendo los criterios propuestos por la OMS se clasificó a los individuos según su IMC en: bajo peso (IMC <18.5), normo peso (IMC=18.5 - 24.9), sobrepeso (IMC= 25.0 -29.9) y obesidad (IMC ≥30).

### Evaluación de la masa ósea

Se llevó a cabo mediante ultrasonografía del calcáneo con el densitómetro CUBA PLUS v4.1.0 (McCue Ultrasonics Limited, Compton, Winchester, UK). El parámetro de mineralización ósea que facilita el densitómetro es el coeficiente de atenuación de la banda ancha (BUA: Broadband ultrasond Atenuattion) medido en dB/MHz. La evaluación ósea mediante ultrasonografía es una técnica usada en pediatría por su reproductibilidad (99%), precisión (error del 1-2%), rapidez (5-10 min) y ser libre de radiaciones ^26^. 

### Ingesta alimentaria

La ingesta alimentaria de nutrientes se estimó mediante un recordatorio de 72 h. Posteriormente fue analizado con el programa informático Nutriber^®^ versión1.1.5. Para valorar la ingesta de micronutrientes, se consideraron ingestas normales teniendo en cuenta el grupo de edad estudiado una ingesta de calcio entre 900 y 1,200 mg/día ^27^, una ingesta de fibra entre 20 y 30 mg/día y una ingesta de fósforo entre 600 y 800 mg/día ^28^.

### Actividad física

La evaluación de la actividad física se realizó mediante el cuestionario Internacional de Actividad Física (IPAQ) ^29^. Es un instrumento validado para estimar la prevalencia de participación en actividad física de la población. La unidad de medida del cuestionario es el MET (Equivalente metabólico= 3.5 mL de O_2_/kg de peso/min), determinándolo de la siguiente manera: intensidad de la actividad física x minutos x días a la semana. La clasificación de los individuos se realizó en función de los MET obtenidos en niveles de actividad física bajo, moderado y alto.

### Análisis estadístico

Las variables numéricas se expresaron como media ± desviación estándar (SD) y las variables nominales como porcentajes y frecuencias. Las diferencias en la ingesta dietética según el sexo se evaluaron a través de una prueba t independiente. La prueba de chi-cuadrado se utilizó para determinar las diferencias entre las variables categóricas. Las diferencias de los valores de BUA según las categorías de IMC y el nivel de actividad física se determinaron mediante el análisis de la varianza (ANOVA). El valor de significancia se estableció como p <0.05. Todos los análisis se realizaron con SPSS versión 20.0 (SPSS, Chicago, IL, EE. UU.).

## Resultados

### Características antropométricas, actividad física y masa ósea

La edad media de los sujetos fue 20.4 ±2.2 años. Los parámetros antropométricos, de actividad física y de mineralización ósea de la población de estudio en función del sexo se muestran en la Tabla 1. El 70% de los individuos (70.1% mujeres y 69.9% hombres) tienen un IMC dentro de los parámetros de normalidad. La prevalencia de obesidad y sobrepeso en el total de la población es del 20% frente a un 10% que corresponde a sujetos con bajo peso. 

**Tabla 1:** Características antropométricas, actividad física y masa ósea en la población de estudio.

	Mujeres (n= 117)	Varones (n= 83)
	Media ±DE	Media ±DE
Edad (años)	20.10 ±2.06	20.76 ±2.40
Talla (m)	1.64 ±0.06	1.76 ±0.07
Peso (kg)	58.75 ±10.22	73.69 ±12.27
Masa grasa (kg)	14.43 ±7.09	11.53 ±6.26
Masa magra (kg)	44.32 ±3.73	62.12 ±7.73
**IMC (%)**		
Bajo peso	17.0 (14.5)	3.0 (3.6)
Normo peso	82.0 (70.1)	58.0 (69.9)
Sobrepeso/Obesidad	18.0 (15.4)	22.0 (26.5)
**Nivel de actividad física** (%)		
Bajo	21.0 (17.9)	7.0 (8.4)
Moderado	64.0 (54.7)	34.0 (41.0)
Alto	32.0 (27.4)	42.0 (50.6)
**Ultrasonografía calcáneo**		
BUA (dB/MHz)	82.8 ±16.3	97.9 ±16.5

DE: desviación estándar; Coeficiente de atenuación de la banda ancha (BUA).

El 14% de los sujetos presentaron un nivel bajo de actividad física, un 49.0% moderado y un 37.0% alto. El 50.6% de los varones presentaron niveles altos de actividad física frente al 24.7% en mujeres. Similarmente, se destaca que sólo un 8.4% de los varones presentó niveles bajos de actividad física versus un 17.9% en mujeres.

El valor medio obtenido tras la valoración de la mineralización ósea mediante ultrasonografía fue 89.07 ±17.93 dB/MHz. La media del valor del BUA fue inferior en las mujeres frente a los varones (82.83±16.29 vs 97.87±16.45), encontrándose diferencias estadísticamente significativas (*p*= 0.013) (dato no mostrado).

### Hábitos nutricionales

Se llevó a cabo un análisis de los hábitos nutricionales en función del sexo atendiendo a macronutrientes (hidratos de carbono, proteínas y lípidos) y micronutrientes (calcio, fósforo y fibra) (Tabla 2). No se han encontrado diferencias estadísticamente significativas en los patrones nutricionales entre hombres y mujeres (dato no mostrado). Cabe destacar los desequilibrios existentes dentro de la ingesta calórica así como de los macro y micronutrientes. Las dietas predominantes en la población de estudio son las de carácter hiperprotéico e hiperlipídico y en ambos sexos la ingesta de calcio y de fibra es inferior a las recomendaciones. Por el contrario, se observa de forma generalizada una alta ingesta de fósforo.

**Tabla 2:** Hábitos nutricionales en función del sexo.

Ingesta	Mujeres		Varones	
	n	%	n	%
**Energía**				
Dieta Hipocalórica	63	55.3	57	68.7
Dieta Normocalórica	20	17.5	9	10.8
Dieta Hipercalórica	31	27.2	17	20.5
**Hidratos de carbono**				
Dieta baja en Hidratos de carbono	61	52.6	39	47.0
Dieta normal en Hidratos de carbono	17	14.7	19	22.9
Dieta alta en Hidratos de carbono	38	32.8	25	30.1
**Proteínas**				
Dieta Hipoprotéica	2	1.7	1	1.2
Dieta Normoprotéica	40	34.5	28	33.7
Dieta Hiperprotéica	74	63.8	54	65.1
**Grasas**				
Dieta Hipolipídica	44	37.9	27	32.5
Dieta Normolipídica	20	17.2	26	31.3
Dieta Hiperlipídica	52	44.8	30	36.1
**Calcio**				
Dieta baja en calcio	74	63.8	47	56.6
Dieta normal en calcio	24	20.7	18	21.7
Dieta alta en calcio	18	15.5	18	21.7
**Fósforo**				
Dieta baja en fósforo	3	2.6	1	1.2
Dieta normal en fósforo	13	11.2	3	3.6
Dieta alta en fósforo	100	86.2	79	95.2
**Fibra**				
Dieta baja en fibra	77	66.4	47	56.6
Dieta normal en fibra	18	15.5	23	27.7
Dieta alta en fibra	21	18.1	13	15.7

No se observaron diferencias estadísticamente significativas en función del género.

### Índice de masa corporal, nivel de actividad física y masa ósea 

Los valores de mineralización ósea en función de IMC (bajo peso, normopeso y sobrepeso/obesidad) y del nivel de actividad física (bajo, moderado y alto) se muestran en la Tabla 3. Se han encontrado diferencias estadísticamente significativas en los valores de BUA entre los sujetos con bajo peso y normopeso (*p*= 0.001), normopeso y sobrepeso/obesidad (*p*= 0.001) y bajo peso y sobrepeso/obesidad (*p* <0.001) en mujeres. Sin embargo, en el caso de los varones no se evidencian diferencias significativas. Respecto al nivel de actividad física, en los varones se observan diferencias estadísticamente significativas entre los sujetos con un nivel bajo y moderado (*p*= 0.021), y bajo y alto (*p*= 0.015) de actividad física. 

**Tabla 3:** Masa ósea en función del IMC y el nivel de actividad física.

	Mujeres (n= 117)			Varones (n= 83)		
	**IMC**					
	Bajo peso (n= 17)	Normo peso (n= 82)	Sobrepeso /Obesidad (n= 18)	Bajo peso (n= 3)	Normo peso (n= 58)	Sobrepeso/Obesidad (n= 22)
	Media ±DE	Media ±DE	Media ±DE	Media ±DE	Media ±DE	Media ±DE
BUA (dB/Mhz)	^a^69.47±13.71	^b^82.65±13.73	^c^101.38±18.57	87.33±16.80	96.10±14.95	102.82±19.85
	**Nivel de actividad física**					
	Bajo (n= 21)	Moderado (n= 64)	Alto (n= 32)	Bajo (n=7 )	Moderado (n= 34)	Alto (n=42)
BUA (dB/Mhz)	79.33±15.94	84.48±17.30	81.81±14.36	^d^87.00±9.16	98.79±17.97	^e^98.93±15.73

DE: Desviación estándar; BUA: Coeficiente de atenuación de la banda ancha.

^a^Bajo peso vs normopeso *p* <0.001.

^b^Normopeso vs sobrepeso/obesidad *p*<0.001

^c^Sobrepeso/obesidad vs bajo peso *p* <0.001.

^d^Bajo vs moderado *p* <0.05.

^e^Bajo vs alto *p* <0.05.

## Discusión

El perfil de los adultos jóvenes sobre algunos de los factores ambientales que determinan la adquisición de una adecuada mineralización ósea es poco conocido. En el presente estudio vealúa el estado nutricional, los hábitos nutricionales, la actividad física y los valores de mineralización ósea de 200 adultos jóvenes. Nuestros resultados evidecian que el IMC y el nivel de actividad física se asocian significativamente con la salud ósea en una población de adultos jóvenes, mostrando que son factores predictores del estado ósea en edades tempranas. 

Los datos del estado nutricional obtenidos muestran una tendencia al sobrepeso y obesidad superior en los varones (26.5%) frente a la mujeres (15.4%). Por el contrario, la prevalencia de bajopeso es mayor en las mujeres en comparación con los hombres (14.5% versus 3.6%). Si se comparan los datos anteriores con los publicados en la Encuesta Nacional de Salud publicada en España ^30^ se puede observar un patrón similar mostrando como la prevalencia de obesidad y el sobrepeso es superior en varones. De manera similiar, también se muestra una tendencia al bajopeso por parte de las mujeres frente a los varones.

En relación a la actividad física, los hombres presentan mayores niveles de actividad física alta que las mujeres, 50.6% versus 27.4%. Respecto al nivel de actividad física moderada, ambos sexos presentan valores similares (41.0% en varones frente a 54.7% en mujeres). Sin embargo, las mujeres presentan notoriamente un nivel de actividad física bajo (mujeres 17.9% versus hombres 8.4%). Cabe destacar que el número de individuos que presentan valores de actividad física alta resultan elevados (37.6%) en comparación con los datos mostrados por la Encuesta Nacional de Salud que evidencian que cuatro de cada 10 personas se declara sedentaria ^30^.

Los patrones nutricionales observados en la población de estudio evidencian ingestas superiores a las recomendadas de lípidos y proteínas en detrimento de los hidratos de carbono. De igual forma se constata que, en su mayoría, los adultos jóvenes mantienen una ingesta calórica insuficiente respecto a sus necesidades. Nuestros resultados son coincidentes con los obtenidos en estudios previos en poblaciones de adultos jóvenes españoles ^31^^-^^34^. 

El nivel medio de masa ósea en adultos jóvenes (89.07 ±17.93) es similar al evidenciado en estudios previos ^35^^-^^37^. En función del género, se muestran diferencias estadísticamente significativas en los valores de mineralización ósea en función del sexo, presentando las mujeres niveles significativamente más bajos que los varones. Las diferencias en los valores de masa ósea entre hombres y mujeres han sido previamente evidenciadas en estudios anteriores ^37^^,^^38^, además de coincidir con la mayor prevalencia de osteoporosis en mujeres en la edad adulta ^39^. Este hallazgo podría evidenciar que la influencia de factores hormonales y genéticos en la osteoporosis comienza a actuar en edades tempranas. 

En consonancia con estudios previos ^(^^23^^,^^24^^,^^31^, en el análisis de la mineralización ósea en función de IMC se observan valores mayores conforme asciende la categoría ponderal (bajo peso, normo peso y sobrepeso/obesidad) en ambos sexos. La falta de diferencias significativas en los varones puede ser debido al menor tamaño muestral o a las diferencias existentes en nuestra población entre ambos sexos en cuanto al estado nutricional.

Además, en línea con estudios previos ^6^^,^^7^, los resultados obtenidos evidencian diferencias estadísticamente significativas en los niveles de masa ósea en función del nivel de actividad física (bajo, moderado y alto) en los varones, mostrando valores más altos del BUA en el calcáneo conforme mayor es el nivel de actividad física. El hecho de que sólo se evidencien diferencias estadísticamente significativas en los hombres probablemente sea debido a las notables diferencias en cuánto al nivel de actividad física entre ambos grupos, presentando las mujeres valores medios de actividad física inferiores.

Respecto a las limitaciones del estudio, cabe destacar su diseño de corte transversal que impide establecer relaciones de causalidad. Además, otra posible limitación sería la utilización de un cuestionario auto-administrado para la evaluación del nivel de actividad física. No obstante, es importante destacar que el IPAQ es una herramienta ampliamente validada para la estimación de la actividad física ^29^. Por último, el hecho de que la población de estudio sólo se incluyeran adultos jóvenes podría limitar la generalización de los resultados a otras poblaciones. 

## Conclusión

Nuestros resultados muestran que el índice de masa corporal y el nivel de actividad física se asocian significativamente con la salud ósea en una población de adultos jóvenes, evidenciando la importancia del fomento de estilos de vida saludables como estrategia para la prevención temprana de la osteoporosis. Sería de gran interés la realización de futuros trabajos que investigaran el rol de los factores genéticos en la masa ósea en la adultez temprana ya que se ha establecido que pueden influir hasta en un 80% en la adquisición del PMO.
